# Estimating the impact of virus testing strategies on the COVID-19 case fatality rate using fixed-effects models

**DOI:** 10.1038/s41598-021-01034-7

**Published:** 2021-11-04

**Authors:** Anthony Terriau, Julien Albertini, Emmanuel Montassier, Arthur Poirier, Quentin Le Bastard

**Affiliations:** 1 GAINS, Le Mans University, 72000 Le Mans, France; 2 GATE, University of Lyon 2, 69000 Lyon, France; 3 MiHAR Lab, University of Nantes, 44000 Nantes, France; 4 Department of Emergency Medicine, Nantes University Hospital, 44000 Nantes, France; 5 LED, Paris 8 University, 93526 Saint Denis, France

**Keywords:** Infectious-disease diagnostics, Virology, Public health

## Abstract

The SARS-CoV2 has now spread worldwide causing over four million deaths. Testing strategies are highly variable between countries and their impact on mortality is a major issue. Retrospective multicenter study with a prospective database on all inpatients throughout mainland France. Using fixed effects models, we exploit policy discontinuities at region borders in France to estimate the effect of testing on the case fatality rate. In France, testing policies are determined at a regional level, generating exogenous variation in testing rates between departments on each side of a region border. We compared all contiguous department pairs located on the opposite sides of a region border. The increase of one percentage point in the test rate is associated with a decrease of 0.0015 percentage point in the death rate, that is, for each additional 2000 tests, we could observe three fewer deaths. Our study suggests that COVID-19 population testing could have a significant impact on the mortality rate which should be considered in decision-making. As concern grows over the current second wave of COVID-19, our findings support the implementation of large-scale screening strategies in such epidemic contexts.

## Introduction

Since reported in late December 2019 from the Hubei province in China, the severe acute respiratory syndrome coronavirus 2 (SARS-CoV2) has now spread worldwide with more than 191 million confirmed cases by July 2021^1^. The outbreak reached Europe via Italy at the end of February and quickly affected the entire continent, making Europe the epicenter by mid-March. The World Health Organization (WHO) declared SARS-CoV2 a pandemic in mid-March 2020. Highly effective vaccines have recently become available, and while waiting for a herd immunity, successive epidemic waves and regional outbreaks have led to the saturation of many healthcare systems. To prevent such a situation in the early stage of the epidemic, governments have implemented various public health measures such as mobility restrictions, social distancing, or large-scale testing strategies. On March 16th, the head of the WHO pronounced in favor of massive population tests^2^. Yet, there is a growing debate about the impact of mass testing on mortality rates^3^. We have observed strong differences in testing rates between countries. For example, South Korea, Germany, and Iceland, have undertaken broad screening policies and now report low case fatality rates. On the contrary, countries like Spain or France have restricted access to diagnostic tests for inpatients or healthcare workers and now report higher mortality rates^4^. Unfortunately, cross-country comparisons (or cross-state comparisons in the case of the US) are complicated owing to great heterogeneity in terms of methods for counting deaths and tests, reporting systems, endowments for medical centers, and lockdown strategies.

In contrast, France has a relatively centralized health system. Beginning March 17, 2020, a strict lockdown approach was instituted for all regions^5,6^. At the same time, the Health Regional Agencies (ARS) were given autonomy in terms of screening strategies implementation. This generated unprecedented variations in testing intensity across regions, largely exogenous at the departmental level, an administrative subdivision of a region. We took advantage of this early period of the pandemic, when no vaccine was available, to evaluate the impact of testing strategies for covid-19 on mortality. The aim of our study was to evaluate the effect of differences in testing strategies in the various regions vis-à-vis the COVID-19 case fatality rate by comparing all adjacent department pairs in France that border a given region. Screening policies and mortality rates could be related to the fact that testing allows authorities to detect and isolate infected people and to prevent them from transmitting the virus. It also enables early treatment, thereby increasing the chances of survival^7^.

## Results

### Regional variations in testing and case fatality rates

**Figure 1:**
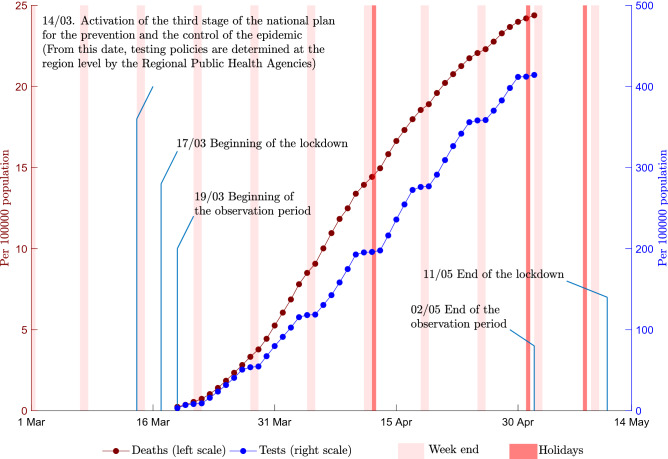
Cumulated number of SARS-CoV2 tests and deaths related to COVID-19, France, March–May 2020. Number of tests and deaths are expressed for 100,000 inhabitants. The cumulated number of deaths are those observed in hospitals during the study period. The cumulated number of tests are the RT-PCR tests performed in non-hospital laboratories during the study period.

Between March 19th and May 2nd 2020, the number of RT-PCR for SARS-CoV2 detection increased from 2,093 tests to 265 662 in mainland France representing an average by department of 8.24 tests per 100 000 inhabitants/day (Fig. 1, Table 1). There was a wide disparity in the number of tests performed by region. When ranking regions by quintiles, computed on the basis of the average number of RT-PCR tests divided by the number of patients admitted to the hospital for COVID-19, we observed that 13.35 tests were performed per 100 000 inhabitants/day in regions of the upper quintile and 4.41 tests per 100 000 inhabitants/day in regions of the lower quintile (−8.94, 95% CI: −14.65 to −3.23; *P* = 0.0035).

**Table 1:** Number of SARS-CoV2 tests, deaths, hospital and ICU admissions in metropolitan regions, France, March–May 2020 (n = 12).

Variables	Overall regions	5st quintile regions	1st quintile regions	95% confidence intervals	*P*-values
Number of tests, mean (SD)	8.24 (7.28)	13.35 (11.07)	4.41 (4.84)	[− 14.65; − 3.23]	0.0035
Number of positive tests, mean (SD)	1.01 (1.05)	0.8 (0.83)	0.74 (0.90)	[− 0.63; 0.51]	0.8300
Number of deaths, mean (SD)	0.46 (0.46)	0.18 (0.11)	0.66 (0.58)	[0.20; 0.77]	0.0021
Hospital length of stay in days, mean (SD)	30.29 (26.99)	15.53 (8.82)	39.97 (32.23)	[8.49; 40.4]	0.0045
ICU length of stay in days, mean (SD)	5.90 (4.92)	3.48 (1.95)	6.85 (5.45)	[0.63; 6.12]	0.018
Number of hospital admissions, mean (SD)	2.77 (2.24)	1.59 (0.87)	3.61 (2.76)	[0.65; 3.40]	0.0059
Number of ICU admissions, mean (SD)	0.42 (0.34)	0.26 (0.15)	0.50 (0.39)	[0.04; 0.44]	0.019

Sample means and standard deviations (SD) are reported for all French departments and those for 5th and 1st quintile regions. Values are reported per 100,000 inhabitants-day and computed for each department. Confidence intervals and p-values are calculated at a 95% confidence level for the 1st and the 5th quintile for means comparison using Student’s t-test. Quintiles are computed on the basis of the average number of RT-PCR tests divided by the number of patients admitted to the hospital for COVID-19. Data are available at https://www.sae-diffusion.sante.gouv.fr/.

*ICU* intensive care unit, *SARS-CoV-2* severe acute respiratory syndrome coronavirus 2, *SD* standard deviations.

**Figure 2:**
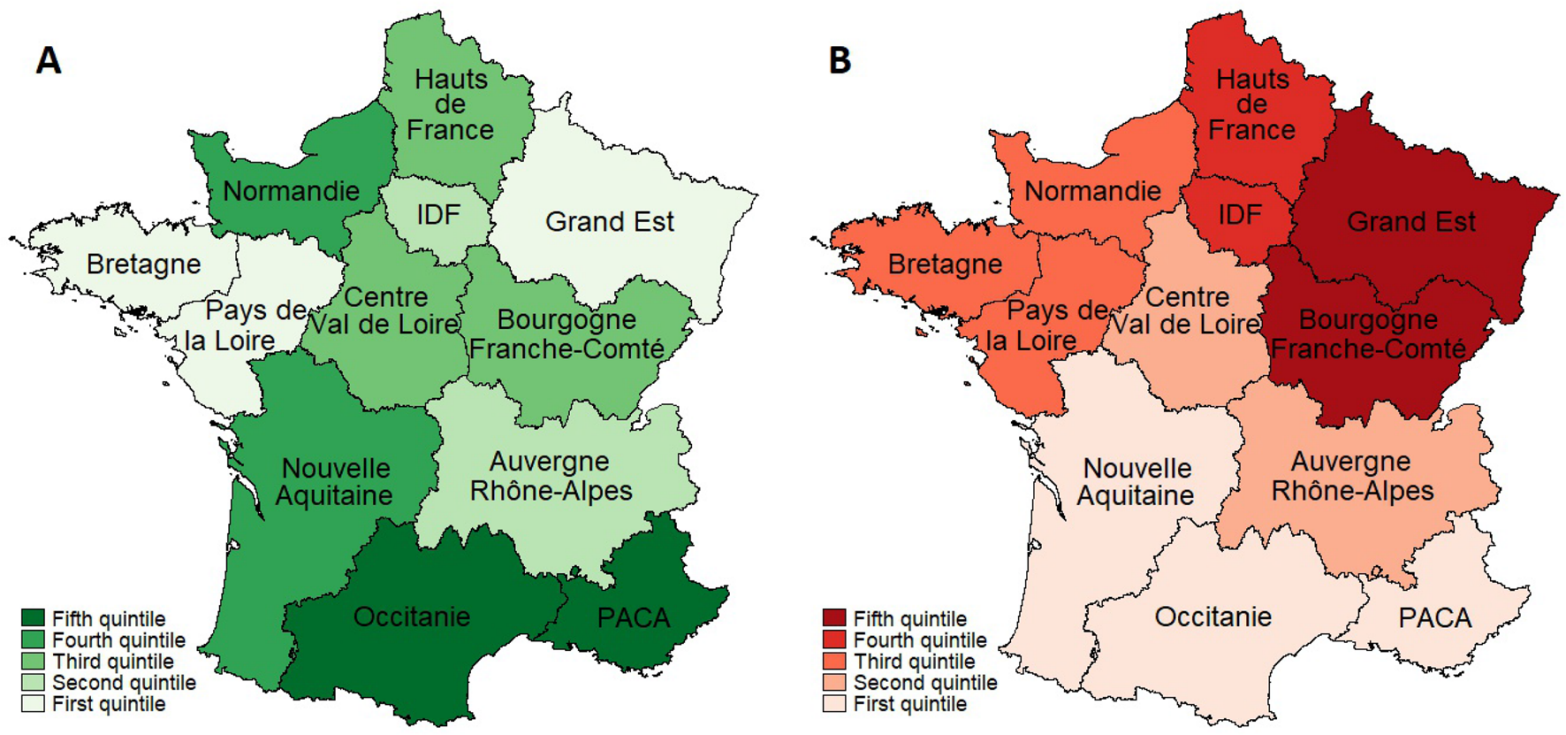
Tests and death rates in metropolitan regions, France, March–May 2020. Panel (**A**): Test rate: number of RT-PCR tests divided by the number of patients admitted to hospitals for COVID-19. Panel (**B**): Death rate: number of deaths in hospitals due to COVID-19 divided by the number of patients admitted to hospitals. We used shapefiles for regions and departments to construct the maps and compute the contiguity matrix. PACA is in the top 20% of regions that test more and in the bottom 20% of regions that have the lowest fatality ratios. Data are freely available at https://www.sae-diffusion.sante.gouv.fr/.

There were 15,378 cumulative deaths over the study period, representing an average of 0.46 deaths per 100,000 inhabitants/day (Table 1). The number of deaths increased quickly from the beginning of the study period (Fig. 1). Mortality was significantly higher in regions of the lower quintile, with 0.64 deaths per 100,000 inhabitants/day, than in regions of the upper quintile, with 0.17 deaths per 100,000 inhabitants/day (0.48, 95% CI: 0.20 to 0.77; *P* = 0.0021). Testing and mortality were not homogeneous throughout the country. The autonomy given to Regional Public Heaths Agencies generated differences in testing rates across regions and strong discontinuities at region borders (Fig. 2).

We noted a total of 95 551 hospital admissions and 15 862 ICU admissions, corresponding to 2.77 admissions per 100,000 inhabitants/day and 0.42 admission per 100 000 inhabitants/day respectively (Table 1). This represented 1,086,821 hospitalization days and 220,693 ICU hospitalization days over the study period. The number of hospitalization days and hospital admissions were significantly higher in regions of the lower quintile than in regions of the higher quintile (15.53 hospitalization days per 100 000 inhabitants/day in regions of the upper quintile versus 39.97 hospitalization days per 100,000 inhabitants/day in regions of the lower quintile (24.45, 95% CI: 8.49 to 40.4; *P* = 0.0045). Moreover, there were more ICU hospitalization days and admissions in the regions of the lower quintile (3.48 hospitalization days per 100,000 inhabitants/day in regions of the upper quintile versus 6.85 hospitalization days per 100,000 inhabitants/day in regions of the lower quintile, (3.37, 95% CI: 0.63 to 6.12; *P* = 0.018). Other descriptive variables are reported in Table S1.

### Estimates using all-departments sample models

The department’s fixed effect captures time-invariant heterogeneity across departments. This includes sociodemographic variables (such as the structure of age, socio-economic status, or gender in the population), but also many variables related to health facilities (number of hospitals, medical density or medical devices). We added time-varying confounding factors in models (b) and (c), model (b) including ICU occupancy rate, and model (c) was also controlled for the rate of positive tests. The first variable controls for the capacity of hospitals to treat patients at different stages of the COVID-19 epidemic while the second controls for selection bias. Our baseline estimates revealed that a 1 percentage point increase in the tests/hospitalizations ratio leads to a statistically significant decrease in the case mortality rate by slightly less than 0.0013 percentage point (95% CI, −0.000843 to −0.001681) (Table 2).

**Table 2:** Estimation of the effect of SARS-CoV2 testing strategy on the mortality rate using fixed effect model in the all-departments sample, France, March–May 2020. (n = 94).

	Model		
	Baseline	Control for ICU occupancy rate	Control for ICU occupancy rate and rate of positive tests
Test rate [CI 95%]	− 0.001262 [− 0.000819; − 0.001706]	− 0.001162 [− 0.000726; − 0.001599]	− 0.001262 [− 0.000843; − 0.001681]

The models analyzed 45 periods, with 12 regions, and 94 departments. Results are calculated in percentage points with 95% confidence intervals (CI 95%). When controlled for ICU occupancy and rate of positive tests, the model using all-departments sample shows an increase of 1 percentage point in the tests/hospitalizations ratio leads to a statistically significant decrease in the case mortality rate of 0.0013 percentage point.

*CI* confidence interval, *ICU* intensive care unit.

### Estimates using contiguous border department-pairs sample models

We then concentrated our analysis on contiguous border department-pairs which exploits policy discontinuities at region borders. As for the all-department sample models, we added time-varying confounding factors to model (e), ICU occupancy rate, and model (f) was also controlled for the rate of positive tests. Our estimates revealed that a 1 percentage point increase in the tests/hospitalizations ratio leads to a statistically significant drop of case mortality rate by 0.0015 percentage point (95% CI, − 0.000441 to − 0.002526) (Table 3). Putting these numbers into perspective means that for each additional 2000 tests, we observe three less deaths.

**Table 3:** Estimation of the effect of SARS-CoV2 testing strategy on the mortality rate using fixed effect models in the contiguous border department-pairs sample, France, March–May 2020. (n = 237).

	Model		
	Baseline	Control for ICU occupancy rate	Control for ICU occupancy rate and rate of positive tests
Test rate [CI 95%]	− 0.001507 [− 0.000177; − 0.002837]	− 0.001415 [− 0.000150; − 0.002680]	− 0.001483 [− 0.000441; − 0.002526]

The models analyzed 45 periods, with 12 regions, 69 departments, and 237 department-pairs. Results are calculated in percentage points with 95% confidence intervals (CI 95%). When controlled for ICU occupancy and rate of positive tests, the model using contiguous border department-pairs sample shows an increase of 1 percentage point in the tests/hospitalizations ratio leads to a statistically significant decrease in the case mortality rate of 0.0015 percentage point.

*CI* confidence interval, *ICU* intensive care unit.

## Discussion

SARS-CoV-2 outbreak is one of the gravest public health emergencies of international concern in decades. Countries have implemented various measures mostly based on mobility restriction, social distancing, and regional or national lockdowns. All of these public health measures are aimed at "flattening the curve" of the infected cases to limit avoidable mortality due to overburdened healthcare systems. In the present study, we evaluated the effect of COVID-19 testing strategy on the mortality rate in France during the national lockdown. We took advantage of the difference in screening intensity between French regions. We first estimated the effect of testing on the case fatality rate using the canonical fixed effects model and the all-departments sample and found that an increase in the screening rate of 1 percentage point enables the mortality rate to decrease by nearly 0.0015 percentage point. We confirmed our results by estimating the fixed effects model using the contiguous border department-pairs, which compared contiguous French departments sharing regional borders.

To the best of our knowledge, no large randomized controlled trial has been implemented to investigate the effect of testing on the case fatality rate. When RCT are difficult to implement or unethical, natural experiments are one of the best alternatives. The principle of natural experiments is to mimic the existence of treatment and control groups using an instrumental variable that induces a change in the explanatory variable but has no direct effect on the outcome. However, in the case of the COVID-19 epidemic, finding a suitable instrument is a difficult task. In the absence of a randomized controlled trial or natural experiments, many researchers are trying to approximate using standard methods such as linear regression, logistic regression, or propensity scores. However, such methods are subject to omitted-variable bias, leading to severe bias in estimating the effects of the variables that are included. Consequently, causal inference via statistical adjustment represents a poor alternative to randomized experiments. In such a context, panel data models represent the best way to control for heterogeneity and to improve causal estimation^8^. We used a fixed effects model because it represents a powerful tool for longitudinal data analysis^9^. However, such a model requires substantial differences between treatment intensities across entities and time to obtain precise estimates. Our data meet these conditions: (i) no region has the same test rate path as other regions over the period considered; (ii) the test rate varies greatly across regions and time, whether due to policy choices or availability of tests. Methods based on regional controls and policy discontinuities have several advantages: (i) contiguous border departments are relatively similar, in particular with regard to health trends which are of major importance in the context of an epidemic; (ii) testing policy is determined at the region level and is largely exogenous from the point of view of a department, which rules out potential reverse causality^8^. Using a standard fixed effect model, we can establish whether there is an association between tests and mortality. However, at this stage, we cannot interpret the statistical correlation as the causal effect of tests on mortality. The first reason is that the association may be due to "reverse causality", i.e., a causal effect of mortality on regions' testing policy. As shown by Dube and Huang, using infra-regions geographic area (the department in our case) allows minimizing the issue of reverse causality^8,10^. We follow their approach by carrying out a second set of estimates at the department-level based on discontinuity at region borders. In this second set of estimates, it is much less likely that mortality in a particular department can influence testing policy at the region level. Another reason that may explain the association between tests and mortality is the "omitted variables bias", i.e. the existence of variables that affect both mortality and testing policy. Fixed-effect models allow controlling for all unobservable variables that are invariant over time. However, our estimates may be influenced by time-varying heterogeneity. In particular, our results may be affected by the stage of the COVID-19 pandemic in a particular area. To deal with this issue, we run several alternative specifications including time-varying variables to capture the severity of the outbreak in each geographic area. All specifications provide relatively similar results. However, we cannot rule out the possibility that our estimates may be influenced by unobservable time-varying variables. For the sake of transparency and replicability, we have chosen to use only publicly available data. The natural counterpart is the small number of time-varying variables at our disposal. To capture the intensity of the epidemic in each geographical area (department), test and death rates were expressed for the number of hospitalizations.

Until we have achieved herd immunity, the only way to prevent an unrestrained scenario is to control the spread of SARS-CoV-2. This is a challenging task because asymptomatic infected patients spread the virus. The literature has reported an alarming proportion of asymptomatic infected cases^11^. Epidemiological data from the Diamond Princess cruise ship revealed that only 18% of the positive cases had no symptoms^12^. Two hospitals in New York implemented universal testing for SARS-CoV-2 with nasopharyngeal swabs in women who were admitted for delivery, and revealed that nearly 90% of the patients who were positive for SARS-CoV-2 on admission reported no symptoms^13^. Overall population screening in Iceland revealed that only 57% of participants with positive tests presented COVID-19 symptoms^14^. This proportion could even be higher owing to false negatives^15^. Large-scale testing is part of a strategy to limit the transmission of the virus and the WHO recommends rapid diagnosis and isolation of cases in combination with rigorous tracking and precautionary self-isolation of close contacts. Several authors have supported the implementation of mass screening policies^3,16,17^. Mass screening could positively impact the case fatality rate in different ways. First, unfocused testing, i.e. not limited to symptomatic subjects, could improve the monitoring of the progress of the epidemic and facilitate decision-making by health authorities. The use of "case definition", given the limited knowledge of the new disease, probably resulted in a low sensitivity to detect infected subjects, resulting in a delayed perception of the progression of the epidemic^18,19^. Screening strategies are subject to the availability of tests which indirectly shapes epidemic curves, as has been observed in the USA^20,21^. Second, mass screening may also provide early identification of infected subjects and rapid implementation of isolation measures. Early reports from Wuhan suggest that public health interventions combining universal symptoms surveys, traffic restriction and home quarantine were temporarily associated with increased control of the outbreak^22,23^. A model from Singapore suggests that quarantining infected individuals and their family members, school closure and workplace distancing could reduce the progression of the epidemic but is associated with a significant economic cost^24^. Review from the Cochrane database concludes that quarantine is important in reducing the number of cases but is dependent on screening strategies^25^. This supports that public health decisions should be as focused as possible in order to limit the negative impact on the economy and society^26^. The importance of rapid diagnosis and case identification and isolation will be of utmost importance with the end of lockdowns.

Our results suggest that screening may significantly reduce the case fatality rate during a lockdown period. First, testing enables to speed up the treatment of infected patients, thereby increasing the chances of cure. Second, it allows to detect and isolate infected people and to prevent them from transmitting the virus. There are some limitations to our results. First, our results are dependent on the completeness and accuracy of the reported number of deaths related to COVID-19 and our results may have been impacted by regional under-reporting. Second, our findings focused on France and it would be very hazardous to suppose that they apply to other countries because their exposition to COVID-19 is different, they have adopted different strategies, and have different health structures. However, our findings provide useful lessons for policymakers around the world on how to manage disease outbreaks in the future. Third, one may suspect that our estimates could be affected by time-varying regional public health measures. If various components of regional public health policy change simultaneously (testing, physical distancing, masks, isolation or contact tracing), regressions that include testing changes but exclude changes in other components of the regional public health policy may be biased. In France, most public health measures (physical distancing, isolation, contact tracing, wearing a mask, etc.…), were implemented at the national level over the period considered. Therefore, it is quite unlikely that our results could be affected by variance in the implementation of public health measures across regions. Fourth, to provide further evidence on this relationship, it would be worth applying this methodology to other countries for which such data are available and in which testing policies are sufficiently heterogeneous across geographical areas. Fifth, the data on tests collected by the French Public Health Agency are those put together by private laboratories and do not include those from public hospitals. This represents an important share of tests (between half and two thirds) and we cannot rule out the possibility that this unobservable amount of screening activity may affect our results. Finally, our study cannot quantify the respective contribution of the treatment provided to screened and infected individuals or the lower dissemination of the virus that results from quarantining policies. Thus, by focusing on the early stages of the pandemic our study excludes potential biases induced by herd immunity and mass distribution of effective vaccines, which could reduce the burden of testing on mortality. Indeed, only 4.5% of the French population had been infected at the end of the study period and vaccination campaigns did not begin until early 2021^27^.

## Conclusions

Large scale COVID-19 screening policies were significantly associated with a decrease in the case fatality rate in France. While concern is growing over the current second wave of SARS-CoV2, these results support the implementation of mass screening strategies and could provide important information for decision-makers in the fight against future pandemics.

## Materials and methods

### Study design

We conducted a retrospective study with a prospective database including all patients who were admitted to hospitals and afterwards discharged, all deaths and the total number of tests performed to screen for COVID-19 infection (Reverse Transcriptase-Polymerase Chain Reaction, or RT-PCR) by out-of-hospital medical laboratories^28^. The sample covers the period from March 19 to May 2, 2020, which corresponds to a lockdown period in France. Anonymized patient data was provided by the Department of Public Health and are available at https://www.data.gouv.fr/. We merged this dataset with information on hospital occupancy rates for intensive care units (ICU) published by the French Ministry of Health and available at https://www.sae-diffusion.sante.gouv.fr/. Sociodemographic data were extracted from the National Institute of Statistics and Economic Studies and are available at https://statistiques-locales.insee.fr/.

### Data selection and modelling procedures

We propose a novel approach to assess the impact of testing strategies on mortality rates that exploits policy discontinuities at region borders and contiguous department pairs that are located on opposite sides of a region border. This methodology has been used in an economic setting to evaluate the effects of the minimum wage on earnings and employment in the US^8^.

We exploited the fact that beginning March 14th, the French government activated the third stage of the national plan for the prevention and the control of the epidemic which involves non-systematic testing of symptomatic individuals. From this date, testing policies have been determined at a regional level by Regional Public Health Agencies. Then, as of March 17th, the national lockdown was declared and social distancing and mobility restrictions have been applied uniformly throughout the country. Our model is based on the hypothesis that local policies on testing strategies have led to a wide disparity in testing intensity between regions. We used a fixed effects model to assess the impact of the number of tests performed over time at a local geographical level (department) on fatality cases. In fixed effects models, subjects serve as their own controls, providing a means for controlling for time-invariant heterogeneity, i.e. all possible characteristics that do not change over time^9,29^. This strategy allow us to analyse the association between the evolution of the number of tests performed and the evolution of COVID-19 related mortality between two paired geographical areas with different test rates.

We used two distinct samples: i) The all-department sample that includes 94 departments distributed across 12 regions; ii) The contiguous border department-pair sample that contains all the contiguous department pairs that straddle a region boundary. Metropolitan France counts 96 departments. We excluded two departments, Haute-Corse and Corse-du-Sud, that are part of a region, Corsica, that does not share any direct border with others. Among the 94 departments, 69 lie along a regional border (https://www.data.gouv.fr). Since each department may belong to several department-pairs, we have a total of 237 distinct department-pairs. Our strategy consisted in comparing all contiguous department pairs sharing a regional border (Fig. S1.) to identify the effect of testing on the case fatality rate. To take into account the local intensity of the epidemic, test rates and death rates were calculated using the number of RT-PCR tests and the number of deaths related to COVID-19 scaled to the number of patients admitted to hospitals, respectively.

### Fixed effects model: estimation strategy

#### Models using the all-departments sample

We first estimated the effect of testing on the case fatality rate using the canonical fixed effects model and the all-departments sample (models (a) to (c)):

$$CFR_{it} = \beta_{1} TEST_{it} + \phi_{i} + u_{it}$$

where $i$ denotes the department, $t$ the time, $CFR_{it}$ is the death rate in department $i$ at time $t$, $TEST_{it}$ represents the test rate in department $i$ at time $t$, $\phi_{i}$ is a department fixed effect, and $u_{it}$ an error term.

The department fixed effect captures time-invariant heterogeneity across departments. This includes sociodemographic variables (such as the structure of age, race, or gender in the population), but also many variables related to health facilities (number of hospitals, medical density or medical devices). We added time-varying confounding factors in models (b) and (c). Model (b) included ICU occupancy rate, and model (c) also controlled for the rate of positive tests. The first variable controls for the capacity of hospitals to treat patients at different levels of disease severity while the second controls for selection bias.

#### Models using the contiguous border department-pairs sample

We then turned to a second identification strategy which exploited policy discontinuities at region borders. To achieve identification, we estimated the following model using the contiguous border department-pairs sample (model (d) to (f)):

$$CFR_{ipt} = \alpha + \beta_{2} TEST_{it} + \mu_{i} + \nu_{p} + \in_{ipt}$$

where $i$ denotes the department, $p$ the department-pair, $t$ the time, $CFR_{ipt}$ is the death rate in department $i$ in department-pair $p$ at date $t$, $TEST_{it}$ represents the test rate in department $i$ in department-pair $p$ on date $t$, $\mu_{i}$ represents a department fixed effect and $\nu_{p}$ a department-pair fixed effect. To account for serial correlation, as well as correlation across department-pairs and along a border segment, we follow the procedure described by Dube et al. to compute correct standard errors^8^. As for the all-department sample, model (e) included ICU occupancy rate, and model (f) was also controlled for the rate of positive tests.

### Statistical analysis overview

Descriptive statistics, figures and results of the estimations were obtained using Stata/MP version 14.2 (Stata Corp). We used a threshold of 0.05 with a two-sided test for statistical significance.

## Supplementary Information

Supplementary Information.

## Data Availability

All data and material are freely available. URLs are specified in the manuscript.

## Code availability

Custom codes are available on request to the authors.
