# Supplementary material for: Estimating the impact of virus testing strategies on the COVID-19 case fatality rate using fixed-effects models
Source: Sci Rep. 2021 Nov 4;11:21650. doi: 10.1038/s41598-021-01034-7 (PMC8569180; doi:10.1038/s41598-021-01034-7)
Supplement: Supplementary file 1 — Supplementary Information. [file 41598_2021_1034_MOESM1_ESM.docx]

Supplementary Materials for

Impact of virus testing strategy on the COVID-19 case fatality rate: estimate using a fixed effects model.

**Authors:** Anthony TERRIAU, PhD, Julien ALBERTINI, PhD, Emmanuel MONTASSIER, MD, PhD, Arthur POIRIER, PhD,Quentin LE BASTARD, MD, MSc*

**This file includes:**

Figs. S1

Tables S1


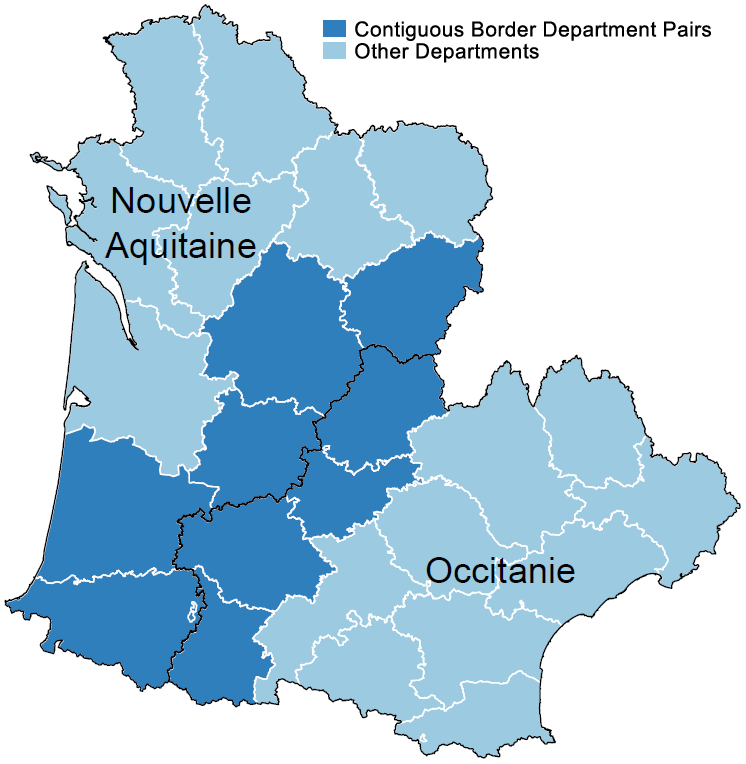


Fig. S1.

**Contiguous border department-pairs.** Example with "Nouvelle Aquitaine" and "Occitanie" regions.

Table S1.

Descriptive statistics of the variables collected in our study. Sample means and standard deviations (SD) are reported for all departments in France and for all contiguous border department-pairs with a full balanced panel of observations.

|  | **All-department Sample** **Mean (SD)** | **Contiguous border department-pair sample** **Mean (SD)** |
| --- | --- | --- |
| **Variables related to individual health** |  |  |
| Number of people hospitalized | 22,59 (39.20) | 21.26 (36.76) |
| Number of tests | 62.80 (143.93) | 57.96 (125.26) |
| Number of positive tests | 8.62 (21.25) | 8.39 (20.32) |
| Number of patients cured | 12.34 (20.90) | 11.56 (19.70) |
| Number of deaths | 3.70 (6.97) | 3.47 (6.50) |
| **Variables related to health facilities** |  |  |
| Number of hospital beds (intensive care unit) | 52.30 (63.33) | 47.30 (55.92) |
| Number of hospital days (intensive care unit) | 16704.23 (21270.02) | 15030.4 (18609.17) |
| Occupancy rate (intensive care unit) | 99.10 (42.19) | 100.09 (44.26) |
| **Sociodemographic variables** |  |  |
| Surface area (in km²) | 52056.77 (22716.04) | 52295.67 (22999.48) |
| Population density | 574.86 (2453.53) | 497.08 (2097.92) |
| % of people aged 65 and over | 21.16 (3.78) | 21.20 (3.93) |
| Household size | 2.18 (0.12) | 2.19 (0.13) |
| Poverty rate | 14.54 (3.02) | 14.45 (2.89) |
| **Sample characteristics** |  |  |
| Number of periods | 45 | 45 |
| Number of departments | 94 | 69 |
| Number of department-pairs | NA | 237 |
| Number of regions | 12 | 12 |
| Observations | 4230 | 21330 |
